# Are patients with pulmonary tuberculosis who are identified through active case finding in the community different than those identified in healthcare facilities?

**DOI:** 10.1016/j.nmni.2016.10.002

**Published:** 2016-11-02

**Authors:** S.T. Abdurrahman, L. Lawson, M. Blakiston, J. Obasanya, M.A. Yassin, R.M. Anderson, O. Oladimeji, A. Ramsay, L.E. Cuevas

**Affiliations:** 1) Federal Capital Territory Tuberculosis and Leprosy Control Programme, Nigeria; 2) Zankli Medical Centre, Nigeria; 3) Nigeria Centre for Disease Control, Abuja, Nigeria; 4) Bingham University, Karu, Nassarawa State, Nigeria; 5) Liverpool School of Tropical Medicine, UK; 6) University of Liverpool, Liverpool, UK; 7) Global Fund to Fight Aids, Tuberculosis and Malaria, Switzerland; 8) Special Program for Research in Tropical Diseases, WHO/TDR, Geneva, Switzerland

**Keywords:** Adults, clinical presentation, close to community services, facility-based services, Nigeria, tuberculosis

## Abstract

The lack of healthcare access contributes to large numbers of tuberculosis (TB) cases being missed and has led to renewed interest in outreach approaches to increase detection. It is however unclear whether outreach activities increase case detection or merely identify patients before they attend health facilities. We compared adults with cough of >2 weeks' duration recruited in health facilities (1202 participants) or in urban slums (2828 participants) in Nigeria. Participants provided demographic and clinical information and were screened using smear microscopy. The characteristics of smear-positive and smear-negative individuals were compared stratified by place of enrolment. Two hundred nine health facility participants (17.4%) and 485 community-based participants (16.9%) were smear positive for pulmonary TB. Community-based smear-positive cases were older (mean age, 36.3 vs. 31.8 years), had longer cough duration (10.3 vs. 6.8 weeks) and longer duration of weight loss (4.6 vs. 3.6 weeks) than facility-based cases; and they complained more of fever (87.4% vs. 74.6%), chest pain (89.0% vs. 67.0%) and anorexia (79.5% vs. 55.5%). Community smear-negative participants were older (mean, 39.4 vs. 34.0 years), were more likely to have symptoms and were more likely to have symptoms of longer duration than smear-negative facility-based participants. Patients with pulmonary TB identified in the community had more symptoms and longer duration of illness than facility-based patients, which appeared to be due to factors differentially affecting access to healthcare. Community-based activities targeted at urban slum populations may identify a different TB case population than that accessing stationary services.

## Introduction

Health services are often inaccessible to ill individuals in the community, and this is particularly pertinent to tuberculosis (TB) in low- and middle-income countries. Despite the scaling up of TB services, it is widely acknowledged there is still a large burden of undiagnosed TB [1], and worldwide, an estimated one third of the 9.5 million cases occurring each year are missed by public health services [2]. An important proportion of these cases are missed because individuals with symptomatic TB fail to seek medical advice for various reasons. The nonspecific presentation of TB, an expectation that symptoms will improve spontaneously and factors such as widespread stigma and misunderstanding about TB can delay or prevent individuals from seeking health services [3], [4]. A further significant barrier to diagnosis is the high transport costs that may arise from multiple visits required for diagnosis [3], [4]. Missed or delayed TB diagnoses may lead to poorer individual outcomes and prolonged transmission, and it limits the impact of TB control measures [5].

Recent years have witnessed renewed interest in the 1978 Alma Ata “Health for All” declaration and the World Health Organization's (WHO) call for universal health coverage. There is thus a need to implement and assess accessible health service delivery to the community, with several models being used to improve access to services and enhance the detection of TB [1], [5], [6]. With effective community services, TB cases could be identified at an earlier stage of disease [5] and larger numbers of patients may be diagnosed, especially from populations underrepresented in stationary health services, such as women, the elderly and the socioeconomically disadvantaged [6]. An increase in detection is likely to be greatest when high-prevalence populations are targeted, and the WHO thus recommends systematic screening of high-risk groups with poor access to healthcare, such as those living in urban slums [5].

There is, however, debate as to whether active community-based interventions increase the number of TB cases detected or whether these cases occurred in patients that would have attended health services eventually but are instead identified at an earlier and less symptomatic stage [5]. The latter assumes that the population detected by community screening is largely the same as that which seeks care at health facilities. Conversely, if TB cases identified by community screening are from a population that would have otherwise remained undiagnosed [1] or that had an intrinsic delay in presentation as a result of differences in healthcare access or health-seeking behaviours [7], then active community screening is likely to have a greater impact on individual prognosis, transmission and total case detection. There are, however, limited data describing whether the profile of TB patients identified in the community differs compared to those identified at health facilities.

We compared the profile of two large groups in the Federal Capital Territory (FCT) of Abuja, Nigeria, who were screened for pulmonary TB (PTB) at health facilities and in the community respectively. On the basis of local anecdote, we hypothesized that community-based PTB patients would have a more prolonged duration of illness and a higher degree of symptomatology.

## Methods

This study took place in the FCT of Nigeria and comprised two substudies screening adults (≥18 years old) for PTB on the basis of cough of more than 2 weeks' duration or other common symptoms associated with TB (night sweats, unexplained fever weight loss or haemoptysis). The studies were conducted between 2010 and 2014. The first study was a multicentre study to evaluate a (then) new scheme called same-day smear microscopy [8]. The FCT arm of this study was conducted in the ambulatory clinics of the FCT Tuberculosis and Leprosy Control Programme (TBLCP) based at five district hospitals within Abuja Metropolitan Area Council (AMAC). Patients with symptoms of TB attend spontaneously or are referred to these TB clinics from other health centres or outpatient services and are routinely screened using smear microscopy. In the same-day scheme, we collected two sputum samples 1 hour apart on the day of first consultation. All adults attending the participating clinics with cough of more than 2 weeks' duration were interviewed by trained nurses and were asked to provide sputum specimens for examination.

The second group consisted of community-based participants from a prospective study exploring whether visiting households of slum settlements surrounding the FCT AMAC to identify adults with symptoms of PTB would increase TB case detection. All slums of the remaining five FCT local government area councils (Abaji, Bwari, Kuje, Kwali and Gwagwalada) were listed and were visited in turn over a period of 18 months. Traditional local leaders were visited to obtain permission to visit the area. Five teams, each consisting of five community health extension workers (CHEWs), one coordinator and a driver, mapped all the houses partnering with local polio programmes and visited the slums. CHEWs then went from house to house to inquire whether there were any residents with cough of more than 2 weeks’ duration and continued canvasing the settlement until all households were visited. Once one or more symptomatic persons in a household was identified, the CHEWs collected demographic and clinical information and obtained two sputum samples 1 hour apart.

Sputum samples in both surveys were transported by study drivers to Zankli TB Research Laboratory the same day as collection. This research laboratory is based at Zankli Medical Centre and is a reference laboratory for the National TBLCP. Smears were examined using light-emitting diode fluorescence microscopy and were graded according to WHO criteria [9]. Demographic and clinical data were collected using a similar structured questionnaire in both surveys. For the purposes of analysis, the two smears collected on the spot 1 hour apart were used for analysis to maintain the comparability of the surveys.

Individuals with positive sputum smears, defined as having at least one acid-fast bacilli in at least one smear, were considered to have PTB. All participants identified with PTB by the surveys were linked to the nearest treatment centre to initiate appropriate therapy. The individuals with negative smears where considered to be largely free from TB for the purpose of analysis. They were advised to seek health services if their symptoms persisted.

Categorical data were summarized using frequency counts and percentages, while continuous data were described using arithmetic means for normally distributed variables (age) and geometric means for skewed variables (e.g. symptom duration). Chi-square and *t* tests were used to assess significant differences between the groups in categorical and continuous variables respectively. Ethical approval for the first study was obtained from the WHO ethics committee as well as the Liverpool School of Tropical Medicine and FCT research ethics committees. Approval for the second study was obtained from the latter two committees. All patients were asked to provide written informed consent to participate.

## Results

A total of 4070 participants with cough of more than 2 weeks’ duration and one or more adequate sputum samples were included. Of these, 1202 (29.5%) were enrolled at healthcare facilities and 2868 (70.5%) from the community. The demographic and clinical features of the facility- and community-based populations are listed in Table 1. As a whole, the population screened in the community had a longer duration of cough (9.9 vs. 4.4 weeks) and a more frequent presence and longer duration of fever, weight loss, chest pain and anorexia compared to health facility–based participants. Two hundred nine (17.4%) of those screened at healthcare facilities and 485 (16.9%) of those screened in the community had positive-sputum-smear PTB.

The demographic and clinical features of the PTB cases are listed in Table 2. Patients with community-based PTB were older than patients with health facility–based PTB, were more likely to be married or in a domestic union and, among the subgroup who volunteered their HIV status, less likely to have HIV infection. Patients with community-based PTB also had a longer duration of cough (10.3 vs. 6.8 weeks) and weight loss (4.6 vs. 3.6 weeks), and they complained more frequently of fever, chest pain and anorexia than patients with facility-based PTB. Patients with health facility–based PTB tended to have higher-grade smears than patients with community-based PTB.

The demographic and clinical features of the presumed largely TB-free participants (negative sputum smear) are listed in Table 3. They had a similar pattern of demographic and clinical differences between sites as the patients with PTB. Within the community-based population, there was no significant difference (p >0.05) in symptom duration between the PTB and TB-free participants. Conversely, within the facility-based population, PTB patients had a significantly longer (p <0.05) duration of all symptoms and a significantly higher frequency of weight loss and anorexia than TB-free participants.

## Discussion

Community-based screening has significant potential to increase TB case detection. There is, however, debate on whether outreach activities are merely identifying individuals earlier and that these patients would eventually seek healthcare, which would thus have a limited effect on total case detection; or if the cases detected in the community represent a different population that, for many reasons, fail to reach the health services. To describe the population reached by an outreach activity in a high-prevalence setting, we compared the characteristics of those screened at stationary healthcare facilities with those actively screened in urban slums.

In both the health facility and community populations studied, sputum smear screening of symptomatic individuals identified a similar high proportion with PTB, reflective of the high prevalence of undiagnosed TB in this setting and in line with the prevalence stated by the national prevalence survey (http://www.who.int/tb/publications/NigeriaReport_WEB_NEW.pdf). The population prevalence and consequent detection rate are important variables when considering the utility of outreach screening programmes [7]. The community-based population as a whole was older, had more symptoms and had a longer duration of symptoms than those screened at healthcare facilities. Furthermore, the community PTB cases and presumed TB-free community-based participants had a similar frequency and duration of symptoms, which suggests that factors common to the whole community group were delaying or preventing them from accessing healthcare. This is not unexpected, as urban slum populations tend to have limited access to health services. Because the community population was more symptomatic as a whole, other comorbidities more common in urban slum populations, such as malnutrition, helminth infection or non-TB bacterial infection, probably contribute to the observed differences in symptomatology between community and facility-based participants and may explain the counterintuitive finding of community-based PTB cases having lower smear grades than facility-based cases. There was a higher prevalence of HIV in the health facility–based patients, which could be partially explained by patients being directed to TB services by the hospital-based HIV clinics. The difference in the prevalence of HIV infection could have contributed to the differences in symptomatology.

PTB cases identified in the community had a higher frequency and a longer duration of symptoms than PTB cases identified at healthcare facilities. This observation contrasts with some previous reports that suggested outreach services may identify less symptomatic cases [10], [11] with a shorter duration of symptoms [12]. This is likely explained by the different approaches used and the populations assessed. We targeted a high-risk subgroup (urban slum residents) of the FCT population for outreach activities, whereas other studies have used community-wide surveys [10], or had community and facility-based populations that were sociodemographically homogenous [12], or described outreach activities in industrialized settings with near-universal access to healthcare services [11].

The increased and prolonged symptomatology of the PTB cases identified in the community population has implications for the impact of outreach activities. Community-based case finding approaches have been shown to increase case detection during the period of implementation, although it has been postulated that it may have a limited effect on overall long-term case detection [13] because, although identified earlier, these patients may have eventually sought care as their symptoms progressed [1]. This is a feasible scenario in areas where the community- and facility-based cases have comparable sociodemographic features and service access. However, the outreach service in this study identified individuals who had been symptomatic for a longer—not shorter—time, which suggests that these patients may not have sought care at a more advanced disease stage and that in this setting community screening may be reaching a different population than those attending stationary health services. Therefore, in this urban slum setting, where individuals likely have socioeconomic constraints on access to healthcare, outreach activities may increase case detection in both the short and long term.

The secondary nature of the data, which limited the number of demographic variables, including income and employment, limited our ability to explore casual pathways for the differences identified between the populations. The unavailability of higher-sensitivity diagnostics such as sputum culture and the absence of other markers of disease severity such as chest radiology also constrained characterization of the populations.

## Conclusion

In this high-prevalence setting, the populations screened for PTB in the community and at stationary health facilities had different clinical characteristics. PTB cases identified by outreach services in urban slums were more symptomatic and had a longer duration of illness than PTB cases identified at health facilities. Outreach activities therefore may reach a different population than stationary services, as differences appear to be due to factors that differentially affect access in the community population at large. This suggests urban slum residents are a population where targeted community-based screening activities could have considerable efficacy for TB case detection.

## Figures and Tables

**Table 1:** Populations screened in community and at healthcare facilities

Characteristic	Community	Healthcare facility	p
Number	2868	1202	—
Age, mean (SD)	39.4 (15.2)	34.0 (10.7)	<0.001
Male, *n* (%)	1447 (51.0)	582 (48.4)	0.142
Marital status, *n* (%)			
Single	610 (21.6)	461 (38.4)	<0.001
Married/union	2075 (73.5)	644 (53.6)	
Widowed	140 (5.0)	97 (8.1)	
HIV status known, *n* (%)	564 (19.7)	675 (56.2)	<0.001
HIV positive, *n* (%)^a^	65 (11.5)	482 (71.4)	<0.001
Cough duration (weeks), mean (SD)	9.9 (2.9)	4.4 (2.3)	<0.001
Fever reported, *n* (%)	2470 (86.3)	817 (68.0)	<0.001
Fever duration (weeks), mean (SD)	3.4 (2.7)	2.1 (2.3)	<0.001
Weight loss reported, *n* (%)	2180 (76.2)	806 (67.1)	<0.001
Weight loss duration (weeks), mean (SD)	4.4 (2.6)	2.5 (2.4)	<0.001
Chest pain reported, *n* (%)	2528 (88.5)	756 (62.9)	<0.001
Chest pain duration (weeks), mean (SD)	3.5 (2.6)	2.1 (2.4)	<0.001
Anorexia, *n* (%)	2087 (73.0)	551 (45.8)	<0.001
Anorexia duration (weeks), mean (SD)	3.2 (2.6)	2.0 (2.2)	<0.001
Submitted two smears, *n* (%)	2811 (98.0)	1187 (98.8)	0.133
PTB, *n* (%)^b^	485 (16.9)	209 (17.4)	0.746

Variable data were missing for some individuals, so percentages were calculated using the number of individuals with available response as the denominator.

PTB, positive for pulmonary tuberculosis.

a Percentage positive of those with known HIV status.

b Individuals who were sputum smear positive for acid-fast bacilli were considered to have PTB.

**Table 2:** Characteristics of PTB cases

Characteristic	Community	Healthcare facility	p
Number	485	209	—
Age, mean (SD)	36.3 (13.6)	31.8 (8.6)	<0.001
Male, *n* (%)	322 (66.8)	125 (59.8)	0.093
Marital status, *n* (%)			
Single	141 (29.4)	94 (45.0)	<0.001
Married/union	326 (68.1)	99 (47.4)	
Widowed	12 (2.5)	16 (7.7)	
HIV status known, *n* (%)	339 (69.9)	127 (60.8)	0.024
HIV positive, *n* (%)^a^	39 (11.5)	83 (65.4)	<0.001
Cough duration (weeks), mean (SD)	10.3 (2.4)	6.8 (2.6)	<0.001
Fever reported, *n* (%)	424 (87.4)	156 (74.6)	<0.001
Fever duration (weeks), mean (SD)	3.4 (2.6)	3.1 (2.5)	0.297
Weight loss reported, *n* (%)	425 (87.6)	180 (86.1)	0.674
Weight loss duration (weeks), mean (SD)	4.6 (2.4)	3.6 (2.5)	0.012
Chest pain reported, *n* (%)	429 (89.0)	140 (67.0)	<0.001
Chest pain duration (weeks), mean (SD)	3.5 (2.6)	3.1 (2.7)	0.292
Anorexia reported, *n* (%)	385 (79.5)	116 (55.5)	<0.001
Anorexia duration (weeks), mean (SD)	3.4 (2.6)	2.9 (2.4)	0.118
Submitted two smears, *n* (%)	477 (98.4)	209 (100)	0.139
Smear grade, *n* (%)			
Scanty	46 (9.6)	24 (11.5)	<0.001
+	166 (34.6)	33 (15.9)	
++	167 (34.8)	55 (26.4)	
+++	101 (21.0)	96 (46.2)	

Variable data were missing for some individuals, so percentages were calculated using the number of individuals with available response as the denominator.

PTB, positive for pulmonary tuberculosis.

a Percentage positive of those with known HIV status.

**Table 3:** Characteristics of tuberculosis-free participants

Characteristic	Community	Healthcare facility	p
Number	2383	993	—
Age, mean (SD)	40.0 (15.4)	34.5 (11.1)	<0.001
Male, *n* (%)	1125 (47.8)	457 (46.0)	0.375
Marital status, *n* (%)			
Single	469 (20.0)	367 (37.0)	<0.001
Married/union	1749 (74.6)	545 (54.9)	<0.001
Widowed	128 (5.5)	81 (8.2)	
HIV status known, *n* (%)	225 (9.4)	548 (55.2)	<0.001
HIV positive, *n* (%)^a^	26 (11.6)	399 (72.8)	<0.001
Cough duration (weeks), mean (SD)	9.9 (3.0)	4.0 (2.2)	<0.001
Fever reported, *n* (%)	2046 (86.1)	661 (66.6)	<0.001
Fever duration (weeks), mean (SD)	3.4 (2.7)	1.9 (2.2)	<0.001
Weight loss reported, *n* (%)	1755 (73.8)	626 (63.0)	<0.001
Weight loss duration (weeks), mean (SD)	4.4 (2.7)	2.3 (2.3)	<0.001
Chest pain reported, *n* (%)	2099 (88.4)	616 (62.1)	<0.001
Chest pain duration (weeks), mean (SD)	3.5 (2.8)	1.9 (2.2)	<0.001
Anorexia reported, *n* (%)	1702 (71.6)	435 (43.8)	<0.001
Anorexia duration (weeks), mean (SD)	3.1 (2.6)	1.8 (2.1)	<0.001
Submitted two smears, *n* (%)	2334 (97.9)	978 (98.5)	0.357

Variable data were missing for some individuals, so percentages were calculated using the number of individuals with available response as the denominator. Individuals with negative sputum smears were considered to be largely tuberculosis free.

a Percentage positive of those with known HIV status.
